# Circulating tumor DNA mutation analysis: advances in its application for early diagnosis of hepatocellular carcinoma and therapeutic efficacy monitoring

**DOI:** 10.18632/aging.205980

**Published:** 2024-07-19

**Authors:** Jing Yang, Na Lin, Miaomiao Niu, Boshu Yin

**Affiliations:** 1 Department of Clinical laboratory, Fourth People’s Hospital of Jinan, Jinan 250031, China

**Keywords:** circulating tumor DNA, hepatocellular carcinoma, early diagnosis, therapeutic efficacy monitoring, high-throughput sequencing

## Abstract

In recent years, the detection and analysis of circulating tumor DNA (ctDNA) have emerged as a new focus in the field of cancer research, particularly in the early diagnosis of hepatocellular carcinoma (HCC) and monitoring of therapeutic efficacy. ctDNA, which refers to cell-free DNA fragments released into the bloodstream from tumor cells upon cell death or shedding, carries tumor-specific genetic and epigenetic alterations, thereby providing a non-invasive approach for cancer diagnosis and prognosis. The concentration of ctDNA in the blood is higher compared to that in healthy individuals or other liquid biopsies from early-stage cancers, which is closely associated with the early diagnosis and comprehensive sequencing studies of HCC. Recent studies have indicated that sequential ctDNA analysis in patients receiving primary or adjuvant therapy for HCC can detect treatment resistance and recurrence before visible morphological changes in the tumor, making it a valuable basis for rapid adjustment of treatment strategies. However, this technology is continuously being optimized and improved. Challenges such as enhancing the accuracy of ctDNA sequencing tests, reducing the burden of high-throughput sequencing on a large number of samples, and controlling variables in the assessment of the relationship between ctDNA concentration and tumor burden, need to be addressed. Overall, despite the existing challenges, the examination and analysis of ctDNA have opened up new avenues for early diagnosis and therapeutic efficacy monitoring in hepatocellular carcinoma, expanding the horizons of this field.

## INTRODUCTION

Hepatocellular carcinoma (HCC) is one of the leading causes of global cancer-related mortality, constituting a significant portion of liver cancer cases. According to the World Health Organization, there are over 840,000 newly diagnosed cases of liver cancer globally each year, with approximately 780,000 deaths, highlighting the persistently high mortality and incidence rates of liver cancer. Particularly in East Asia and Africa, where the prevalence of hepatitis virus infections is high, the incidence of HCC remains elevated [1–3]. The symptoms of liver cancer in the early stages are often subtle, leading to the late detection of many patients who miss the optimal window for surgical intervention. The choice of treatment strategies such as surgical resection, radiofrequency ablation, or liver transplantation is often constrained by tumor size, quantity, location, and the patient’s liver function. While these treatment modalities achieve partial efficacy, the overall cure rate is suboptimal due to significant surgical trauma, potential postoperative complications, and high rates of lesion recurrence [4, 5]. For advanced-stage liver cancer patients opting for targeted therapies such as sorafenib, regorafenib, and cabozantinib, although these treatments extend survival and improve quality of life, they fail to completely eliminate lesions and are plagued by issues of drug resistance [6–8]. In recent years, there has been an increasing emphasis on early diagnosis and therapeutic efficacy monitoring in liver cancer. Conventional imaging studies and the measurement of tumor markers such as AFP (Alpha-fetoprotein), CA125 (Cancer Antigen 125), and CA19-9 remain the mainstays of liver cancer diagnosis. However, the sensitivity and specificity of these methods are significantly limited, failing to meet the demands for early, reliable, and accurate liver cancer diagnosis [9, 10]. Moreover, traditional monitoring methods, such as repetitive imaging examinations, often fall short in detecting minuscule recurrent lesions. Assessing treatment effectiveness typically requires a waiting period for confirmation through imaging or biochemical markers, which may lead to disease progression or the persistence of ineffective treatment strategies [11–13]. Therefore, the exploration of new diagnostic and therapeutic approaches for hepatocellular carcinoma holds paramount clinical significance.

Circulating tumor DNA (ctDNA) comprises fragments of tumor-derived DNA released into the bloodstream upon tumor cell death. These fragments offer insights into the genomic status and dynamic alterations of the tumor [14, 15]. In contrast to conventional tissue biopsies, ctDNA analysis presents a non-invasive avenue for monitoring the epigenetic traits of tumors, encompassing mutations, copy number variations, and methylation patterns [16–18]. This analytical approach not only serves in early screening and diagnosis but also facilitates the assessment of therapeutic efficacy and the prediction of disease recurrence during the treatment process [19, 20]. In tumors, the generation of ctDNA primarily involves the processes of apoptosis and necrosis of tumor cells. When tumor cells die, their DNA is released into the bloodstream, forming ctDNA. Additionally, active tumor cells can also release DNA through the secretion of small vesicles, further increasing the concentration of ctDNA in the blood [21, 22]. Research has revealed that ctDNA can serve as a biomarker for early diagnosis and screening of cancers such as colorectal cancer and non-small cell lung cancer, and it can be detected before the onset of relevant clinical symptoms [23, 24]. Therefore, the detection and analysis of ctDNA can provide molecular characteristic information of the tumor, contributing to early cancer diagnosis and monitoring of therapeutic efficacy [25].

In recent years, significant progress has been made in the study of circulating tumor DNA (ctDNA) with the development of high-throughput sequencing technologies. The analysis of mutation information in ctDNA allows for early cancer diagnosis and monitoring of therapeutic efficacy. Furthermore, the Tagged-Amplicon Deep Sequencing (TAm-Seq) method has been demonstrated as a viable approach for non-invasively identifying gene mutations in blood, capable of amplifying and sequencing large genomic regions, even from a single copy of ctDNA. This method enables the detection of low-frequency mutations, including those in cancer-associated genes such as TP53, EGFR, BRAF, and KRAS [26]. Analysis of mutation sites in ctDNA can also unveil the molecular mechanisms underlying tumor resistance to therapy, thereby providing a basis for developing novel treatment strategies [27]. Moreover, ctDNA can be utilized to detect the presence of residual tumor cells post-surgery or radiation therapy, which holds significant importance in predicting tumor recurrence and guiding subsequent treatment [28, 29].

During the apoptosis process of tumor cells, DNA is fragmented by enzymes and released into the surrounding environment. Additionally, the necrosis of tumor cells can lead to the release of a large amount of DNA into the bloodstream. These released DNA fragments undergo a series of modifications and stabilization processes to form ctDNA. Researchers have also found that factors such as inflammation and angiogenesis in the tumor microenvironment may influence the release and circulation of ctDNA. In recent years, numerous studies have demonstrated the potential of ctDNA mutation analysis in early diagnosis of HCC [30, 31]. Researchers have discovered the presence of common hepatocellular carcinoma (HCC)-associated gene mutations, such as TP53, CTNNB1, and AXIN1, in blood samples from HCC patients [32–34]. These mutations play crucial roles in the occurrence and progression of HCC. Moreover, analysis of ctDNA in the blood samples of 77.3% of HCC patients undergoing anti-PD-1 therapy revealed the presence of >1 genetic alterations, with TP53 being the most common mutated gene, observed in 11 patients [35]. Evaluation of ctDNA mutations can not only be utilized for early screening of HCC but also serves as a vital tool for treatment selection and efficacy monitoring. Specific mutations in ctDNA, such as specific deletions/insertions in the EZH2 gene, can assess the efficacy of chemotherapy in gastric cancer. Similarly, the association between the detection of IDH1 mutations and clinical response offers a new perspective on the treatment of intrahepatic cholangiocarcinoma (IHC). Furthermore, monitoring the mutation spectrum of ctDNA aids in evaluating the diagnostic efficacy of early to intermediate stage non-small cell lung cancer (NSCLC) patients and correlates with tumor size and clinical staging [36].

In this review, we summarized the current understanding of the molecular mechanisms and clinical applications of ctDNA mutation in the occurrence and development of hepatocellular carcinoma, with a focus on its value in early diagnosis and monitoring therapeutic efficacy.

## ctDNA mutation analysis techniques

The mutation analysis of circulating tumor DNA (ctDNA) is an important non-invasive detection method that can be used for early diagnosis and monitoring therapeutic efficacy in hepatocellular carcinoma (HCC) [37, 38]. Over the past few years, significant improvements and innovations have been made in ctDNA mutation analysis methods along with technological advancements. This section will focus on the collection and extraction methods of ctDNA, as well as commonly used ctDNA mutation analysis techniques, including next-generation sequencing (NGS), digital PCR, and TAm-Seq techniques.

Blood sample collection is one of the most commonly used methods for ctDNA collection [39]. ctDNA extraction methods primarily include nucleic acid extraction and ctDNA enrichment. Nucleic acid extraction involves extracting total DNA from plasma or serum, including DNA released from both normal and tumor cells. Common nucleic acid extraction methods include commercial DNA extraction kits and magnetic bead-based methods. On the other hand, ctDNA enrichment methods involve enriching ctDNA from total DNA using specific techniques to enhance detection sensitivity. Presently, methods for ctDNA enrichment primarily comprise Single-Strand Binding Polymerase Chain Reaction (SSB-PCR), probe detection systems based on the IV endonuclease, rolling circle amplification technology, digital PCR, hybrid capture methods, and detection using internal reference nucleic acid probes, as detailed in Table 1.

**Table 1:** Comparison of ctDNA enrichment methods.

**Method**	**Principle**	**Advantages**	**Reference**
SSB-PCR	Specific inhibition chains and primers are designed to selectively enrich low-abundance short DNA strands.	Effective differentiation of fetal wild-type homozygous short DNA, heterozygous short DNA, and mutant homozygous short DNA even at abundances as low as 2%.	[49, 50]
Probe detection based on IV endonuclease	Utilizes the ability of the IV endonuclease to recognize probe vacancies and cleave them, enabling simultaneous detection of multiple mutations at inconsistent detection temperatures through specific probe designs.	Effective discrimination between wild-type DNA and mutant DNA, with effective discrimination among 6 mismatches, demonstrating good universality and accuracy.	[51]
Rolling circle amplification (RCA)	Under the action of polymerases with strand displacement properties, amplification is consistently performed using the initial circular DNA as a template, ensuring that errors generated in each round of amplification are not passed on to the next round.	Capable of enriching trace amounts of cfDNA and amplifying the frequency of low-frequency mutations in ctDNA in allelic genes.	[52]
Digital PCR (dPCR) and hybrid capture	Enriches target DNA by specific probes or antibodies binding to the target DNA sequence, demonstrating superior performance in target enrichment, especially in coverage of target sites across all samples.	Demonstrates superior performance in target enrichment, especially in coverage of target sites across all samples.	[53, 54]
Detection using internal reference probes	Constructs self-sequences as references, unaffected by irrelevant sequences, capable of effectively distinguishing between wild-type and mutant DNA.	Enables quantitative detection of mutation abundance at different concentrations, demonstrating good quantitative limits and accuracy.	[55, 56]

Next-generation sequencing (NGS) is a high-throughput sequencing technology that allows simultaneous analysis of mutations in multiple genes. The advantages of NGS lie in its high sensitivity and specificity, enabling the detection of low-frequency mutations [40]. In the analysis of ctDNA mutations, NGS can be employed for whole exome sequencing, targeted gene sequencing, and quantitative analysis of mutation sites. Through NGS technology, a comprehensive understanding of the tumor mutation profile can be achieved, providing a basis for personalized therapy [41, 42]. Research has shown that Next-Generation Sequencing (NGS) can detect ctDNA mutations with allele frequencies as low as 0.1% and has been successfully applied in ctDNA analysis for various cancer types, including lung cancer, gastric cancer, and intrahepatic cholangiocarcinoma (ICC) [43]. The application of NGS extends beyond the detection of mutations in single genes, encompassing extensive analysis of the entire genome or specific gene regions, thereby uncovering more potential therapeutic targets [44]. Digital PCR (dPCR) is a PCR technology based on the principle of molecular counting, facilitating quantitative analysis of mutation sites in ctDNA. Compared to traditional quantitative PCR techniques, dPCR exhibits higher sensitivity and accuracy. In ctDNA mutation analysis, dPCR can be utilized to detect low-frequency mutations and assess mutation burden. dPCR has been demonstrated to exhibit good accuracy and sensitivity in detecting specific mutations such as KRAS G12D, TP53 C242S, and IDH1 R132C. Additionally, dPCR has been utilized in the development of a novel 5-plex copy number droplet digital PCR (ddPCR) detection platform targeting BRAF or CCND1 copy number amplifications, enhancing the monitoring capabilities for resistance mechanisms [45]. With dPCR technology, precise quantification of ctDNA mutations can be achieved, offering more accurate results for early diagnosis and treatment monitoring of hepatocellular carcinoma [46]. Tagged-amplicon deep sequencing (TAm-Seq) is a deep sequencing method specifically designed for ctDNA, capable of identifying single-copy ctDNA fragments at extremely low allele frequencies. This technology has been applied in ctDNA analysis for various cancers, including high-grade serous ovarian cancer, successfully identifying low-level TP53 mutations and mutations in other key genes [47, 48]. An improved version of TAm-Seq further enhances its ability to detect low-level mutations, making it a crucial tool for monitoring disease progression and treatment response. A comparison of the advantages and disadvantages of three ctDNA mutation analysis techniques is provided in Table 2.

**Table 2:** Comparison of advantages and disadvantages of ctDNA mutation analysis techniques.

**Analysis technique**	**Principle**	**Advantages**	**Disadvantages**
NGS (Next-Generation Sequencing)	Utilizes high-throughput sequencing technology to analyze ctDNA for detecting tumor mutations.	Relatively low cost, wide coverage range; simultaneous detection of multiple genes and mutation types; easy operation, suitable for large-scale sample analysis [57].	Detection sensitivity limited by ctDNA abundance; limited ability to detect low-frequency mutations; requires complex bioinformatics analysis to interpret results [58, 59].
Digital PCR (dPCR)	Divides samples into thousands of microreaction units, where PCR amplification and fluorescence-labeled probe detection are independently performed in each unit, achieving high sensitivity and specificity for specific mutations.	High sensitivity and specificity; capable of detecting extremely low abundance of ctDNA; high accuracy, suitable for Minimal Residual Disease (MRD) detection.	High equipment cost; complex sample preparation process; requires high technical expertise from operators [60].
TAm-Seq (Tagged-Amplicon Deep Sequencing)	Amplifies target regions in ctDNA using specific primers designed, followed by analysis of these amplicons using deep sequencing technology to identify low-frequency mutations.	Capable of detecting mutations with allele frequencies as low as 0.1%; high sensitivity for single-copy ctDNA; provides abundant variant information in a short time [48].	Requires design and optimization of primers for different tumor types and gene loci; may still have detection limitations for extremely low abundance of ctDNA or specific types of mutations; relatively high cost.

In conclusion, ctDNA mutation analysis techniques hold significant value in the early diagnosis and treatment monitoring of HCC. The collection and extraction methods for ctDNA, along with commonly used mutation analysis technologies such as next-generation sequencing (NGS), digital PCR (dPCR), and Tagged-amplicon deep sequencing (TAm-Seq), serve as reliable tools for ctDNA mutation analysis. As technology continues to advance and research deepens, ctDNA mutation analysis techniques are poised to further propel the progress of early diagnosis and treatment monitoring for HCC. They will provide more precise guidance for personalized therapy.

## The application of ctDNA mutation analysis in early diagnosis of hepatocellular carcinoma

In recent years, the analysis of circulating tumor DNA (ctDNA) mutations has emerged as a promising non-invasive detection method, providing new opportunities for the early diagnosis of HCC. This section will focus on the selection and validation of ctDNA mutation markers, a comparison between ctDNA mutation analysis and traditional diagnostic methods, and the clinical application of ctDNA mutation analysis in early HCC diagnosis through case studies.

The selection and validation of ctDNA mutation markers are critical steps in early diagnosis of HCC. Analysis of mutation sites in ctDNA allows for the identification of mutated genes and mutation frequencies associated with HCC [61, 62]. Currently, numerous studies have identified ctDNA mutation markers associated with HCC, such as TP53, CTNNB1, AXIN1, and others [32–34], as shown in Table 3.

**Table 3:** Functional description of ctDNA mutation markers associated with HCC.

**ctDNA**	**Function**	**Source**	**Reference**
TERT Promoter Mutations	Promote cellular proliferation and avoid senescence, associated with HCC development	Hepatocytes	[75, 76]
TP53 Mutations	Regulate cell cycle, DNA repair, and apoptosis	Hepatocytes and surrounding tissues	[32, 77]
CTNNB1 Mutations	Control cell adhesion and signaling, associated with HCC development	Hepatocytes	[32, 78, 79]
ARID1A Mutations	Involved in chromatin remodeling and gene expression regulation, related to liver carcinogenesis	Hepatocytes	[80]
PIK3CA Mutations	Activate signaling pathways for cell growth and survival, implicated in HCC	Hepatocytes	[81, 82]
RAS Mutations	Involved in cell signaling pathways that control cell growth, mutations often found in HCC	Hepatocytes	[83, 84]
ALB-Fusions	Abnormal albumin gene fusions, unique to liver cancer and can be used as a biomarker	Hepatocytes	[85]
APOB Mutations	A pattern of mutations suggesting activity of APOBEC cytidine deaminases, which can contribute to cancer genome mutations	Hepatocytes and immune cells	[86]

The TP53 gene encodes the p53 protein, a tumor suppressor protein capable of inhibiting tumor development by inducing apoptosis or halting cell cycle progression. TP53 mutations typically result in loss of p53 function, thereby promoting proliferation and survival of tumor cells, increasing malignancy and metastatic risk [63]. Studies have shown that TP53 mutations are commonly present in non-small cell lung cancer (NSCLC) and are closely associated with patient prognosis. Additionally, TP53 mutations may also affect patient response to certain treatments such as radiation therapy and chemotherapy [64]. Furthermore, the CTNNB1 gene encodes β-catenin, a cytoskeletal protein involved in regulating the Wnt signaling pathway. CTNNB1 mutations may lead to aberrant activation of the Wnt signaling pathway, promoting proliferation, invasion, and metastasis of tumor cells. However, current research evidence regarding the specific role and clinical significance of CTNNB1 mutations in HCC is relatively limited. The KRAS gene encodes a small G protein involved in various cell signaling pathways, including the insulin-like growth factor-1 (IGF-1) and epidermal growth factor (EGF) signaling pathways. KRAS mutations mainly occur in codons 12 and 13 of exon 2, leading to sustained activation of the KRAS protein, promoting proliferation, invasion, and angiogenesis of tumor cells. KRAS mutations are considered significant adverse prognostic markers in tumors such as colorectal cancer (CRC) and NSCLC [65]. Additionally, KRAS mutations directly impact patient response to anti-EGFR therapy [66].

**Figure 1:**
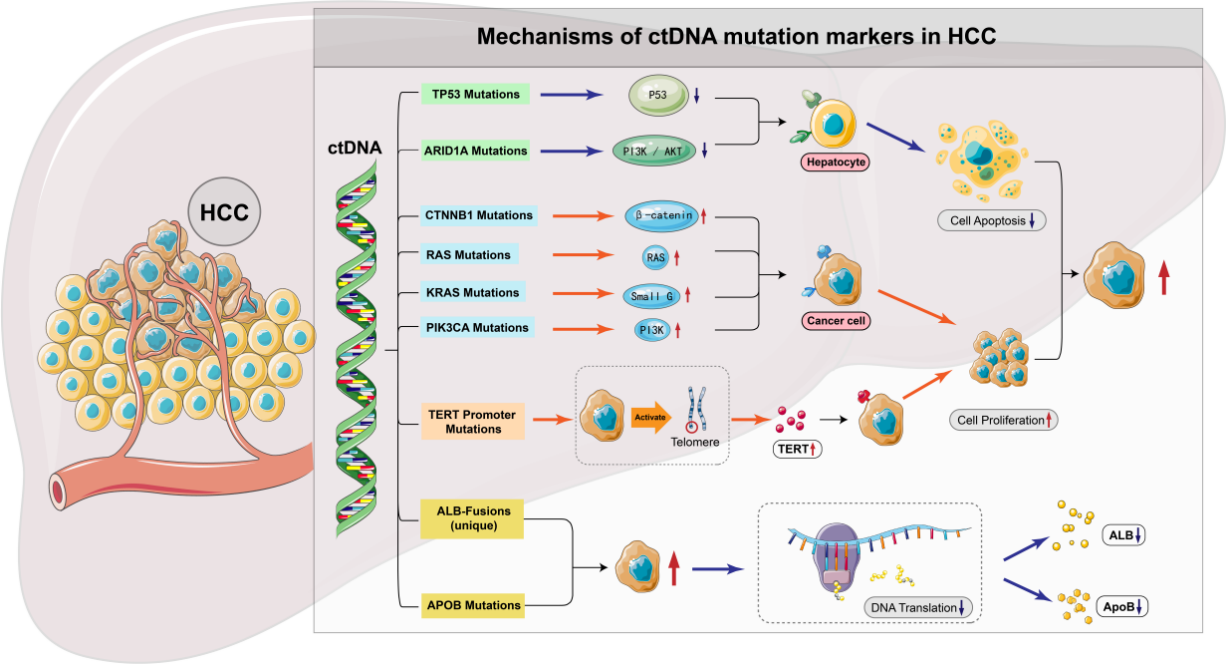
ctDNA mutation markers associated with HCC.

The clinical significance of TP53, CTNNB1, and KRAS gene mutations in HCC patients mainly manifests in their influence on tumor biology, predictive value for treatment response, and impact on prognosis, as depicted in Figure 1. The specific mechanisms of action of these gene mutations suggest the importance of personalized treatment strategies and also offer the potential for the development of new therapeutic targets. Future research needs to further explore the mechanisms of action of these gene mutations in different subtypes of HCC and their impact on treatment outcomes, aiming to provide more precise treatment options for HCC patients.

Compared to traditional diagnostic methods, ctDNA mutation analysis offers several advantages. Firstly, ctDNA mutation analysis is a non-invasive detection method that only requires a blood sample from patients, avoiding the trauma and risks associated with traditional tissue biopsies. Secondly, ctDNA mutation analysis exhibits high sensitivity and specificity, enabling the detection of low levels of tumor DNA and accurate diagnosis even at early stages of the disease. Additionally, ctDNA mutation analysis can provide genetic information about the tumor, such as mutation burden and mutation profile, which can guide personalized therapy [67, 68]. In comparison to traditional tumor markers such as AFP, ctDNA mutation analysis demonstrates higher sensitivity and specificity in early diagnosis of HCC. A study comparing ctDNA mutation analysis with AFP in early HCC diagnosis showed that the sensitivity and specificity of ctDNA mutation analysis were 85% and 92% respectively, while AFP exhibited sensitivity and specificity of 60% and 80% respectively. This indicates that ctDNA mutation analysis can provide more accurate and reliable early diagnosis results [69, 70].

A study analyzing the ctDNA mutation profile of 100 HCC patients identified several mutated genes associated with HCC, such as TP53, CTNNB1, and AXIN1 [71]. Another study found that ctDNA mutation burden in the blood sample of HCC patients was closely related to tumor size, staging, and prognosis, indicating that ctDNA mutation analysis could be used to evaluate patients’ prognosis and treatment response [72]. In addition to early diagnosis, ctDNA mutation analysis can also be utilized to monitor HCC patients’ treatment efficacy. A study analyzing the changes in ctDNA mutation burden after HCC patients received radiation therapy or targeted treatments found that the reduction of mutation burden was closely related to treatment response and survival rate improvement. This indicates that ctDNA mutation analysis could be used to evaluate treatment efficacy and predict the risk of recurrence [73, 74].

Therefore, as an emerging, non-invasive detection method, ctDNA mutation analysis shows great potential in early diagnosis of HCC. Through the selection and validation of ctDNA mutation markers, accurate guidance for individualized treatment and early diagnosis of HCC can be achieved. Clinical studies have already confirmed the potential of ctDNA mutation analysis in early diagnosis and treatment monitoring of HCC. With ongoing development of technology and research, ctDNA mutation analysis will provide more accurate and reliable methods for early diagnosis and treatment monitoring of HCC.

## Application of ctDNA mutation analysis in treatment monitoring of hepatocellular carcinoma

Treatment monitoring is crucial for clinical decision-making in HCC, as it plays a significant role in evaluating treatment efficacy, guiding subsequent treatment strategies, and predicting patient prognosis [87]. The latest advancements in ctDNA mutation assessment in monitoring the therapeutic efficacy of HCC primarily encompass its significance as a tumor biomarker, its application in immunotherapy, its relationship with hepatocellular carcinoma, and its potential in treatment response monitoring. These advancements offer new perspectives and approaches for the diagnosis, treatment, and prognosis assessment of HCC. This section will focus on the association between ctDNA mutation burden and treatment response, the application of ctDNA mutation analysis in targeted therapies, and its utilization in radiation therapy and chemotherapy.

The ctDNA mutation burden refers to the quantity and frequency of mutation sites in ctDNA. Studies indicate a close correlation between ctDNA mutation burden and tumor size, staging, and prognosis. During the course of hepatocellular carcinoma (HCC) treatment, changes in ctDNA mutation burden can reflect treatment efficacy and predict the risk of recurrence [88]. An analysis of HCC patients undergoing radiation therapy or targeted treatment revealed a significant correlation between the reduction in mutation burden and improved treatment response and survival rates [89]. This suggests that ctDNA mutation burden serves as a crucial indicator for evaluating treatment efficacy.

Targeted therapy is a treatment method directed at specific molecular targets, aiming to enhance treatment efficacy and reduce side effects. The application of ctDNA mutation analysis in targeted therapy can assist in selecting suitable targeted drugs and monitoring treatment efficacy. By analyzing mutation sites in ctDNA, the mutational spectrum of HCC patients and the targets for targeted therapy can be identified. A study revealed a close correlation between EGFR mutations in HCC patients and sensitivity to targeted therapy. Through ctDNA mutation analysis, patients with EGFR-positive mutations can be identified and appropriate targeted drugs can be selected for treatment [90]. Another study found a close correlation between BRAF mutations in HCC patients and sensitivity to targeted therapy. Through ctDNA mutation analysis, patients with BRAF-positive mutations can be identified, and suitable targeted drugs can be selected for treatment [91]. Radiation therapy and chemotherapy are common treatment methods for HCC, but there are limitations in monitoring their efficacy. The application of ctDNA mutation analysis in radiation therapy and chemotherapy can provide more accurate and dynamic information on treatment efficacy. An analysis of HCC patients undergoing radiation therapy revealed a significant correlation between the reduction in mutation burden and improved treatment response and survival rates [92]. This indicates that ctDNA mutation analysis can be utilized to assess the efficacy of radiation therapy and predict the risk of recurrence. Another study found a significant correlation between the reduction in ctDNA mutation burden and improved treatment response and survival rates in HCC patients undergoing chemotherapy, further confirming the value of ctDNA mutation analysis in chemotherapy [93].

In summary, ctDNA mutation analysis, as an emerging non-invasive detection method, demonstrates significant potential in the monitoring of treatment efficacy for hepatocellular carcinoma (HCC). The association between ctDNA mutation burden and treatment response suggests that analyzing mutation sites in ctDNA can assess treatment efficacy and predict the risk of recurrence. In targeted therapy, ctDNA mutation analysis can assist in selecting suitable targeted drugs and monitoring treatment effectiveness. In radiation therapy and chemotherapy, ctDNA mutation analysis can offer more accurate and dynamic information on treatment efficacy. As technology continues to advance and research deepens, ctDNA mutation analysis will provide more accurate and reliable methods for monitoring the treatment efficacy of HCC, offering precise guidance for personalized treatment.

## Challenges and future development of ctDNA mutation analysis

As the application of circulating tumor DNA (ctDNA) mutation analysis in the early diagnosis and monitoring of treatment efficacy for hepatocellular carcinoma (HCC) continues to develop, some challenges and future development directions are gradually emerging. This section will focus on the improvement of detection sensitivity and specificity of ctDNA, standardization and calibration of ctDNA mutation analysis, the combined use of ctDNA mutation analysis with other biomarkers, and the prospects of ctDNA mutation analysis in personalized treatment.

In the analysis of ctDNA mutations, the key metrics are the detection sensitivity and specificity [94]. Despite notable progress to date, several challenges persist. Firstly, due to the extremely low abundance of ctDNA in blood, the development of more sensitive detection methods is imperative to enhance sensitivity. Secondly, given the sequence similarity between ctDNA and normal cell DNA, there is a need to further improve specificity to prevent misdiagnosis and errors in judgment. Future research endeavors may explore innovative technologies and approaches, such as amplifying the signal of ctDNA, increasing sequencing depth, and utilizing more specific mutation markers, to enhance the sensitivity and specificity of ctDNA detection. Standardization and calibration of ctDNA mutation analysis are crucial for ensuring accuracy and reproducibility. Standardized methods and procedures need to be established, as the lack of uniformity in experimental conditions and analytical methods across different laboratories and research teams has resulted in poor comparability and consistency of results [25, 62]. Therefore, the establishment of a unified standardization and calibration approach is paramount. This includes standardizing the collection, processing, and storage of samples, as well as experiment conditions and analytical procedures. Additionally, the creation of reference standards and a quality control system is essential. Through standardization and calibration, the reliability and reproducibility of ctDNA mutation analysis can be enhanced, providing more dependable results for clinical applications.

The combined application of ctDNA mutation analysis with other biomarkers holds the potential to further enhance the accuracy and reliability of early diagnosis and treatment monitoring for hepatocellular carcinoma (HCC). For instance, incorporating serum markers such as alpha-fetoprotein (AFP) and hepatocellular carcinoma-related antigen (HCC-RA) can improve the sensitivity and specificity of early diagnosis [95]. Additionally, integrating imaging modalities such as ultrasound, CT, and MRI can provide a more comprehensive overview of tumor information [96]. Future research endeavors should explore the synergistic use of ctDNA mutation analysis with other biomarkers to elevate the accuracy and reliability of diagnosing and monitoring the treatment efficacy of hepatocellular carcinoma. The prospect of ctDNA mutation analysis in personalized therapy lies in tailoring treatment plans based on individual patient differences and the genetic characteristics of tumors. By providing genetic information about the tumor, such as mutation load and mutational lineage, ctDNA mutation analysis serves as a foundation for personalized treatment decisions. Analyzing mutation sites in ctDNA allows for the identification of mutational lineages and targets for targeted therapy. Future studies can further investigate the application of ctDNA mutation analysis in personalized therapy, including the selection of appropriate targeted drugs, monitoring treatment effectiveness, and predicting patient prognosis.

In summary, ctDNA mutation analysis has made significant progress in the early diagnosis and treatment monitoring of hepatocellular carcinoma (HCC). However, several challenges and future directions exist. These include improving the detection sensitivity and specificity of ctDNA, standardizing and calibrating the methods and procedures for ctDNA mutation analysis, exploring the combined application of ctDNA mutation analysis with other biomarkers, and further leveraging the role of ctDNA mutation analysis in personalized therapy. With the continuous advancement of technology and deeper research, it is believed that ctDNA mutation analysis will provide more accurate and reliable methods for the early diagnosis and treatment monitoring of HCC, offering more precise guidance for personalized therapy.

## Conclusion

ctDNA mutation analysis holds significant value in the early diagnosis of hepatocellular carcinoma (HCC). Early diagnosis has been a massive challenge due to the hidden nature and lack of specific symptoms of HCC. However, as a non-invasive detection method, ctDNA mutation analysis can achieve early diagnosis by analyzing mutation sites in ctDNA. By screening and validating ctDNA mutation markers, it is possible to identify HCC-related mutated genes and mutation frequencies, thereby enhancing the accuracy and reliability of early diagnosis. Additionally, ctDNA mutation analysis has important applications in monitoring the treatment efficacy of hepatocellular carcinoma. Treatment monitoring is vital for assessing treatment responses, guiding subsequent treatment strategies, and predicting patient prognosis. By analyzing changes in ctDNA mutation load, treatment effectiveness can be evaluated, and the risk of recurrence can be predicted. Research indicates a close correlation between a decrease in ctDNA mutation load and improved treatment response and survival rates. Therefore, ctDNA mutation analysis can serve as a non-invasive monitoring method, providing more accurate and reliable results for the treatment monitoring of hepatocellular carcinoma.

Furthermore, future research directions and prospects are worth considering. Firstly, there is a need to further improve the detection sensitivity and specificity of ctDNA mutation analysis to enhance the accuracy of early diagnosis and treatment monitoring. Secondly, standardized methods and procedures need to be established to ensure the reliability and reproducibility of ctDNA mutation analysis. Additionally, exploring the combined application of ctDNA mutation analysis with other biomarkers can enhance the accuracy and reliability of the diagnosis and treatment monitoring of hepatocellular carcinoma. Lastly, ctDNA mutation analysis has potential in personalized therapy, enabling the selection of the most suitable treatment plan based on individual patient differences and the genetic characteristics of tumors.

In summary, ctDNA mutation analysis holds significant value in the early diagnosis and treatment monitoring of hepatocellular carcinoma (HCC). By improving the accuracy and reliability of early diagnosis, as well as evaluating treatment effectiveness and predicting patient prognosis, ctDNA mutation analysis can provide more precise personalized treatment guidance for HCC patients. Future research efforts should focus on further advancing the technology and methods of ctDNA mutation analysis, promoting its clinical application, and strengthening multi-center collaboration and large-sample studies to validate its potential in the early diagnosis and treatment monitoring of HCC. It is believed that with continuous technological advancements and in-depth research, ctDNA mutation analysis will provide more accurate and reliable methods for the early diagnosis and treatment monitoring of HCC, leading to a significant improvement in patient survival rates and quality of life.
